# Geographic Distribution and Seasonality of Brown Dog Tick Lineages in the United States

**DOI:** 10.1093/jme/tjac172

**Published:** 2022-11-07

**Authors:** Amber N Grant, Megan W Lineberry, Kellee D Sundstrom, Kelly E Allen, Susan E Little

**Affiliations:** Department of Veterinary Pathobiology, College of Veterinary Medicine, Oklahoma State University, Stillwater, OK, USA; Department of Veterinary Pathobiology, College of Veterinary Medicine, Oklahoma State University, Stillwater, OK, USA; Department of Veterinary Pathobiology, College of Veterinary Medicine, Oklahoma State University, Stillwater, OK, USA; Department of Veterinary Pathobiology, College of Veterinary Medicine, Oklahoma State University, Stillwater, OK, USA; Department of Veterinary Pathobiology, College of Veterinary Medicine, Oklahoma State University, Stillwater, OK, USA

**Keywords:** brown dog tick, *Rhipicephalus sanguineus*, seasonality, temperate, tropical

## Abstract

Two lineages of brown dog ticks (*Rhipicephalus sanguineus* sensu lato (s.l.), Latreille [Acari: Ixodidae]) have been described in North America: temperate and tropical. To characterize the distribution of these lineages across this region and evaluate seasonal activity, a 12S rRNA mitochondrial gene fragment was sequenced from *R. sanguineus* s.l. collected from hundreds of dogs and cats from different locations across 25 of the 50 states from 2018 to 2021. Infestations with temperate lineage predominated (78.5%) and were identified on pets from 20 states, with most (83.5%) from areas with annual mean daily average temperature <20°C. Tropical lineage submissions were less common (19.3%), submitted from 15 states, and most (80.0%) tropical lineage ticks were from areas with an annual mean daily average temperature >20°C. Although travel history was not obtained for all dogs, when tropical lineage infestations were found in colder regions, follow up conversations with veterinarians suggested some of these infestations may have resulted from recent travel of dogs. A limited number (2.2%) of dogs from Arizona and Texas were co-infested with both lineages. Both temperate and tropical lineage ticks were collected from pets in every month of the year. Temperate lineage infestations were primarily collected March through August while tropical lineage infestations were more often collected June through November. These data confirm at least two lineages of *R. sanguineus* s.l. are present in the United States, each predominating in distinct, overlapping geographies, and suggest that peak activity of each lineage occurs at different times of the year.

*Rhipicephalus sanguineus* sensu lato (s.l.), commonly called brown dog ticks, are found worldwide. Unlike most other ixodids, species in this tick group are endophilic and establish thriving indoor infestations (Dantas-Torres 2008, Dantas-Torres 2010). As the colloquial name denotes, brown dog ticks primarily infest dogs, but occasionally parasitize other animals including humans (Dantas-Torres 2008, Nicholson et al. 2010); these ticks are known to transmit numerous canine and zoonotic pathogens and are therefore a concern to veterinary and public health across the globe (Nicholson et al. 2010, Dantas-Torres 2010, Saleh et al. 2021). Organisms known or strongly suspected to be transmitted by *R. sanguineus* s.l. include bacteria (*Anaplasma, Ehrlichia,* spotted fever group *Rickettsia* spp.), protozoa (*Babesia vogeli* Reichenow [Piroplasmida: Babesiidae]*, Hepatozoon canis* [Eucoccidiorida: Hepatozoidae]), and nematodes (*Acanthocheilonema dracunculoides* Cobbold [Spirurida: Onchocercidae], *Cercopithifilaria bainae* Almeida & Vicente [Spirurida: Onchocercidae]) (Olmeda-García and Rodríguez-Rodríguez 1994, Inokuma et al. 2003, Demma et al. 2005, Irwin 2009, Parola et al. 2009, Little et al. 2010, Demoner 2013, Ramos et al. 2014, Little et al. 2021).

Knowledge of the species comprising the *R. sanguineus* s.l. complex is incomplete but continuing to grow as researchers further investigate biologic, genetic, and morphologic variations among its members (Dantas-Torres et al. 2013, Nava et al. 2015, Dantas-Torres et al. 2018, Sanchez-Montes et al. 2021). Currently, *R. sanguineus* s.l. is considered to be an assemblage of as many as 14 to 17 different species belonging to several discreet operational taxonomic units (OTUs), or lineages (Dantas-Torres et al. 2018, Šlapeta et al. 2021). Hybrid mating experiments have shown that although some larvae hatch in some studies, most eggs produced from hybridization between temperate and tropical lineage brown dog ticks are infertile, suggesting they are distinct, reproductively incompatible species (Szabó et al. 2000, Levin et al. 2012). A male brown dog tick of the temperate lineage was described as neotype, leading to assignment of the temperate lineage as *Rhipicephalus sanguineus* sensu stricto (s.s.) (Nava et al. 2018). More recently, the name *Rhipicephalus linnaei*, Audouin [Acari: Ixodidae] was proposed for the tropical lineage (Šlapeta et al. 2021). Both *R. sanguineus* s.s. (temperate lineage) and tropical lineage brown dog ticks have been documented in the United States (Zemstova et al. 2016, Jones et al. 2017, Brophy et al. 2022). Research in this area is ongoing and a consensus on species nomenclature for the tropical lineage in the Americas has not been reached. Accordingly, we use the terms *R. sanguineus* s.s. (temperate lineage) and tropical lineage in the present paper.

Limited data are available regarding the geographic distribution and seasonal activity of *R. sanguineus* s.l. in North America. However, tropical lineages are predominantly collected in regions where the annual mean daily average temperature is >20°C (e.g., Florida, Hawaii, and southern Texas along the Mexico border), while *R. sanguineus* s.s. (temperate) lineage is usually found in cooler, drier climates and thus thought to be more widely distributed on the continent (Eremeeva et al. 2011, Zemstova et al. 2016, Jones et al. 2017). Historic seasonality patterns were largely reported before awareness of distinct species within the complex and thus are best viewed as *R. sanguineus* s.l. of unknown lineage. Under laboratory conditions, the life cycle of *R. sanguineus* s.l. is most efficient at temperatures of 20–35°C and relative humidity of 35–95% (Koch 1982, Dantas-Torres 2010). Provided canine hosts are present, *R. sanguineus* s.l. activity may be sustained year-round indoors. In warmer climates these ticks also persist outdoors although oviposition, egg hatching, and molting of immature stages are less likely to occur in lower temperatures (Koch 1982, Dantas-Torres 2010). Phenology of the different lineages and response to abiotic climatic clues (e.g., temperature, humidity) appears to vary, affecting diapause and seasonality of activity (Labruna et al. 2017). The present study was conducted to more fully characterize the geographic distribution and begin to assess seasonality of the two predominant lineages of *R. sanguineus* s.l. active in the United States.

## Materials and Methods

*Rhipicephalus sanguineus* s.l. used in this study were collected from dogs and cats through a national survey in the United States as previously described (Saleh et al. 2019). In total, 214 veterinary practices from all 50 states were enrolled in the study and provided materials for tick submission; submissions also were accepted from additional, nonenrolled practices. Briefly, invitations were sent to veterinary clinics in each state until 2–4 clinics had been enrolled in each state. All submissions were voluntary, and no attempt was made to normalize tick collection efforts across the country. In addition, we did not limit enrollment and thus some municipal areas were represented by multiple clinics while enrollment was sparse in other areas. Each enrolled practice was provided a tick submission kit consisting of instructions, submission forms, tick containers, forceps, and prepaid mailing envelopes. A study website also provided instructions and an email link was available to address any questions (Saleh et al. 2019). Veterinary staff were asked to remove all ticks identified on a dog or cat, regardless of species, and place the ticks in a hard-plastic container with a tightly fitting lid; occasionally tick submissions were made using serum tubes or other rigid, sealed containers. The container with ticks was then sealed in a plastic bag, a submission form completed, and both specimen and submission form shipped to our laboratory. Geographic location of the veterinary practice where ticks were collected (City, State) and date ticks were removed from the infested animal were recorded. A map of annual mean daily average temperatures was obtained from the United States Climate Atlas, National Centers for Environmental Information (NOAA 2022). Ticks received were identified morphologically to species and stage following standard keys (Clifford et al. 1961, USDA 1976) and then preserved in 70% ethanol at −20°C until further testing. Only ticks identified morphologically as *R. sanguineus* s.l. were included in the present study; other tick species were also received and are reported elsewhere (Saleh et al. 2019).

Total nucleic acid was extracted from *R. sanguineus* s.l. in each submission as previously described (Jones et al. 2017). Briefly, adult *R. sanguineus* s.l., or immature *R. sanguineus* s.l. when no adults were submitted, were dissected and all internal contents were removed and extracted with a commercial kit (illustra blood genomicPrep Mini Spin Kit, Cytiva, Piscataway, NJ; or QIAamp DNA Blood Kit, Qiagen, Germantown, MD) following the manufacturer’s instructions. A 12S rRNA mitochondrial gene segment (340–370 bp) was amplified from each specimen as previously described (Szabó et al. 2005); positive amplicons were purified using a commercial kit (Wizard SV Gel and PCR Clean-Up System, Promega, Madison, WI) and sequenced using Sanger method at Oklahoma State University Molecular Core Facility (Stillwater, OK). Each sequence was compared to all available 12S rRNA gene sequences of *R. sanguineus* s.l. in GenBank and to a curated subgroup of reference sequences from confirmed lineages (KC243786–KC243807, OM985276–OM985391, OM177220, OM177221) with *R. sanguineus* s.l. lineage assigned when identity to reference sequences varied by <1% (Dantas-Torres et al. 2013, Brophy et al. 2022, Mumcuoglu et al. 2022). To determine if pets were co-infested with multiple lineages, sequence was obtained from up to 10 individual ticks submitted from a given pet. Confidence intervals (95%) of all proportions were calculated by modified Wald (Agresti and Coull 1998). Seasonality was evaluated with Chi square tests and a *P* value significance threshold of 0.05.

## Results

### Hosts and Tick Stages

**Fig. 1.**
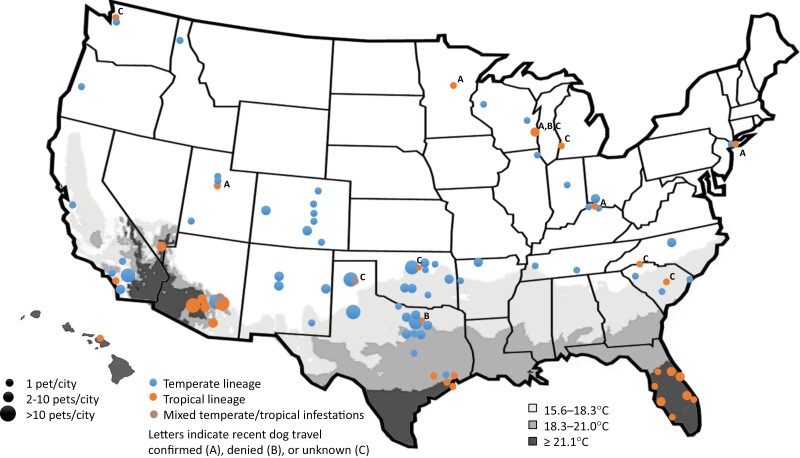
Geographic location of pets infested with *Rhipicephalus sanguineus* sensu stricto (temperate) (blue) and tropical (orange) lineages of *Rhipicephalus sanguineus* sensu lato across the United States, 2018–2021. Grayscale shading indicates areas with warmer annual mean daily average temperature. Superscripts indicate travel history status of dogs with tropical lineage ticks identified in cooler climates.

**Fig. 2.**
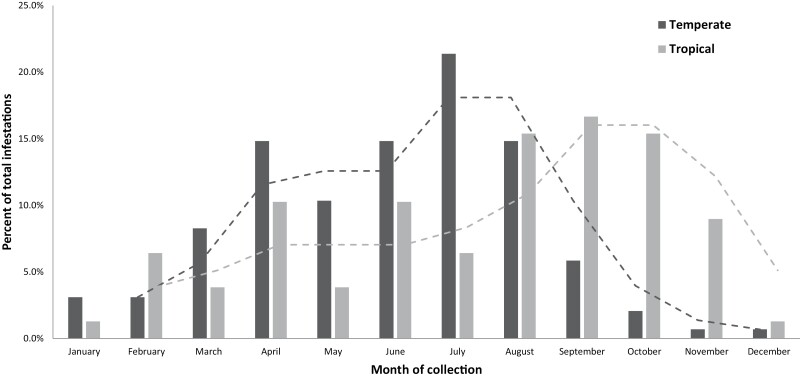
Monthly proportion of *Rhipicephalus sanguineus* sensu stricto (temperate) and tropical lineages of *Rhipicephalus sanguineus* sensu lato infestations from pets. Dashed lines show two month rolling averages for each lineage.

**Fig. 3.**
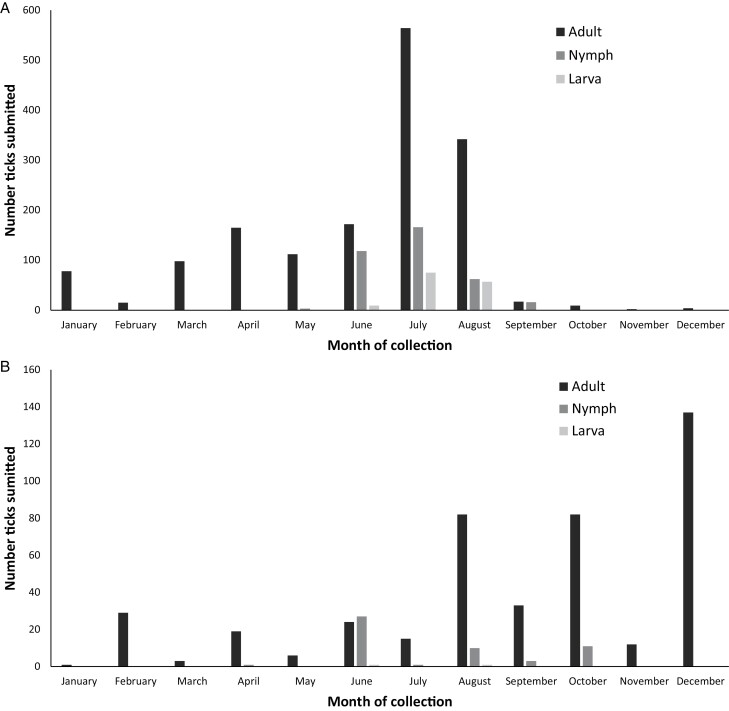
Total number and stages of *Rhipicephalus sanguineus* sensu lato collected from pets each month. Excludes dogs co-infested with both *R. sanguineus* s.s. (temperate) and tropical lineage ticks and one dog infested with >4,000 brown dog ticks. Please note differences in y-axis scale reflecting differences in number of submissions of each lineage. (a). *Rhipicephalus sanguineus* sensu stricto (temperate lineage) collected from pets each month. (b). Tropical lineage ticks collected from pets each month.

In total, 3,065 *R. sanguineus* s.l. (2,086 adults, 814 nymphs, 165 larvae) were collected from 377 individual pets, including 370 dogs and 7 cats. One additional dog, a 7-year-old intact male Basset Hound from Amarillo, Texas, USA, had a very large number of *R. sanguineus* s.l. submitted (>4,000) and was excluded from some analysis to avoid skewing data. Excluding the single dog with thousands of ticks, dogs had a range of 1−393 *R. sanguineus* s.l. ticks collected from a single animal with an average of 8.2 (95% CI 5.4–11.1; median = 2.5). Cats had a range of 1–7 *R. sanguineus* s.l. ticks collected from a single animal with an average of 2.0 (95% CI 0–4.1; median = 1). Adult *R. sanguineus* s.l. were submitted from most infested pets (357/378; 94.4%, CI 91.6–96.4), and male ticks (*n* = 1,122) outnumbered female ticks (*n* = 964; *X*^*2*^ = 11.967, df = 1, *P* = 0.0005). Larvae or nymphs of *R. sanguineus* s.l. were collected from 57/378 (15.1%, 95% CI 11.8–19.1) pets, including from which only nymphs or larvae were submitted. A total of 17/371 (4.6%; 95% CI 2.8–7.3) dogs had more than 25 *R. sanguineus* s.l. Brown dog ticks were submitted from pets in 25/50 (50%) states in the United States (Fig. 1; Table 1) and collected from pets in all 12 months (Figs. 2, 3a, and b).

**Table 1.** Total number and stages of each lineage of *Rhipicephalus sanguineus* sensu latosubmitted from dogs or cats from each state

Lineage	Hosts (no.)		Ticks (no.)				
	Dogs	Cats	Female	Male	Nymph	Larva	Total
*Rhipicephalus sanguineus* sensu stricto (temperate lineage)							
Arkansas	7	–	9	18	–	–	27
Arizona	46	–	69	64	7	–	140
California	28	1	59	79	32	5	175
Colorado	12	–	41	29	98	65	233
Idaho	1	–	3	4	–	–	7
Illinois	1	–	1	2	–	–	3
Indiana	1	–	1	–	2	–	3
Kentucky	1	–	1	–	–	–	1
North Carolina	2	–	2	1	–	–	3
New Mexico	15	2	58	129	23	55	265
New York	0	1	1	–	–	–	1
Ohio	2	–	2	–	–	–	2
Oklahoma	36	2	113	92	27	–	232
Oregon	1	–	1	1	–	–	2
South Carolina	3	–	–	1	32	5	38
Tennessee	2	–	8	14	–	–	22
Texas	112	1	297	462	144	11	914
Utah	3	–	2	3	–	–	5
Washington	1	–	5	7	–	–	12
Wisconsin	2	–	1	1	–	–	2
Total temperate	276	7	674	907	365	141	2,087
Tropical lineage							
Arizona	30	–	140	136	42	2	320
California	1	–	–	1	–	–	1
Florida	19	–	30	33	–	–	63
Hawaii	1	–	–	–	7	–	7
Kentucky	1	–	1	–	–	–	1
Michigan	1	–	1	–	–	–	1
Minnesota	1	–	1	1	–	–	2
Nevada	2	–	3	–	–	–	3
New York	1	–	3	2	–	–	5
Oklahoma	1	–	6	–	–	–	6
South Carolina	2	–	1	1	–	–	2
Texas	5	–	57	20	3	–	80
Utah	1	–	–	–	1	–	1
Washington	1	–	–	1	–	–	1
Wisconsin	3	–	3	2	–	–	5
Total tropical	70	0	246	197	53	2	498
Mixed infestations (*R. sanguineus* s.s. [temperate lineage] and tropical lineage)							
Arizona	7	–	15	11	18	–	44
Texas	1	–	1	1	17	–	19
Total mixed	8	0	16	12	35	0	63
**Overall total**	**354**	**7**	**936**	**1,116**	**453**	**143**	**2,648** ^*a*^

^*a*^Excludes ticks from one dog from Texas infested with >4,000 *R. sanguineus* s.s. (temperate lineage) and from 16 dogs with 417 ticks (34 adults, 361 nymphs, 22 larvae) for which sample quality precluded lineage identification.

### Hosts and Tick Lineages

All ticks included in the present study were confirmed to be *R. sanguineus* s.l. by morphology. Additional tick species were also submitted but were not included. A single *R. sanguineus* s.l. was available for sequencing from each of 154 pets (154/362; 42.5%; 95% CI 37.5–47.7), while more than one *R. sanguineus* s.l. (*n* = 2–10) was available for sequencing from each of 208 pets (208/362; 57.5%; 95% CI 52.3–62.4). Amplification and sequencing of a 12S rRNA gene fragment from 1 to 10 ticks from each pet (1,183 total ticks) revealed 284/362 (78.5%; 95% CI 73.9–82.4) pets were infested with only *R. sanguineus* s.s. (temperate lineage) ticks, including 277 submissions from dogs and all 7 submissions from cats. Only tropical lineage ticks were identified from 70/362 (19.3%; 95% CI 14.915.6–23.7) dogs. Both lineages were identified on 8/362 dogs (2.2%; 95% CI 1.1–4.4). Of the dogs with only *R. sanguineus* s.s. (temperate lineage), 4.3% (12/277; 95% CI 2.4–7.5) had >25 ticks submitted. Of the dogs with only tropical lineage, 5.7% (4/70; 95% CI 1.8–14.2) had >25 ticks submitted. Extracted nucleic acid of ticks collected from 16 additional pets infested with *R. sanguineus* s.l. did not amplify despite repeated attempts.

### Variation within Lineages

In total, 960 ticks had 12S rRNA sequences consistent with temperate lineages with most (957/960; 99.7%; 95% CI 99.0–99.9) identified as *R. sanguineus* s.s. (temperate lineage), also referred to as *Rhipicephalus* sp. II. Testing multiple ticks (*n* = 2–10) collected from the same pet resulted in more than one *R. sanguineus* s.s. (temperate lineage) sequence in ticks from 78/175 (44.6%; 95% CI 37.4–52.0) pets. All sequences were >99% identical to those previously reported for *R. sanguineus* s.s. (temperate lineage) ticks, including KC243802 (*n* = 493), OM985340 (*n* = 445), and KC243803 (*n* = 19). Sequence >99% identical to a distinct ‘temperate’ lineage referred to as *R. sanguineus* sp. I or southeastern Europe lineage *Rhipicephalus* sp. (OM177221) was identified in three ticks collected from three different dogs, including two dogs infested with several additional ticks with 12S rRNA sequence of *R. sanguineus* s.s. (temperate lineage), and one dog infested with a single tick with 12S rRNA sequence consistent with southeastern Europe lineage *Rhipicephalus* sp. I. (Dantas-Torres et al. 2013, Brophy et al. 2022, Mumcuoglu et al. 2022).

In total, 233 ticks had 12S rRNA sequences consistent with tropical lineage with most (223/233; 95.7%; 95% CI 92.2–97.7) >99% identical to those reported from dogs worldwide (e.g., GenBank KC243786). Ten ticks from a single dog from Florida had 12S rRNA sequence identical to that reported from tropical lineage ticks from Brazil (GenBank KC243787) (Dantas-Torres et al. 2013). No mixed tropical lineage sequences were identified on individual infested dogs.

### Geographic Distribution of Collections

*R. sanguineus* s.l. were submitted from a total of 25 states; *R. sanguineus* s.s. (temperate lineage) ticks were submitted from 20 states and tropical lineage ticks were submitted from 15 states (Fig. 1; Table 1). Mixed infestations of *R. sanguineus* s.s. (temperate lineage) and tropical lineage were identified on dogs in two states, including 4 dogs from the same household in Arizona, 3 additional dogs from Arizona, and 1 dog from Texas. Most (237/284; 83.5%; 95% CI 78.7–87.3) *R. sanguineus* s.s. (temperate lineage) infestations were from areas where the annual mean daily average temperature is <20°C although this lineage was also identified on several dogs at or near the edge of this temperature range in southern California, Arizona, and Texas (Fig. 1). A distinct southeastern Europe lineage originally referred to as ‘*Rhipicephalus* sp. I’ was identified from three dogs, including two dogs from New Mexico and one from Idaho, each with unknown travel history. *R. sanguineus* s.s. (temperate lineage) ticks were collected from cats in California, New Mexico, New York, Oklahoma, and Texas.

Most (56/70; 80.0%; 95% CI 69.1–87.8) tropical lineage infestations were identified on dogs in areas where the annual mean daily average temperature is >20°C (e.g., Hawaii, and southern regions of Arizona, California, Florida, Nevada, and Texas), but several (14/70; 20.0%; 95% CI 12.2–30.9) dogs infested with tropical lineage were identified in cooler regions, including Kentucky, Michigan, Minnesota, New York, northern Texas, Oklahoma, South Carolina, Utah, Washington (state), and Wisconsin (Fig. 1). When tropical lineage ticks were identified from an unexpected locale, an attempt was made to contact the submitting veterinarian to inquire about recent travel history of the dog; 5 confirmed the dog had recently traveled to a warmer region, 2 denied any recent travel, including one dog from northern Texas and one dog from Wisconsin, and the remaining 7 did not respond or were unable to obtain travel information from the client (data not shown).

### Seasonality of Collections

Both lineages were collected every month of the year (Figs. 2 and 3; Table 2). Most pets from which *R. sanguineus* s.s. (temperate lineage) were collected (239/282; 84.8%) presented from March to August (*X*^*2*^ = 136.3, df = 1, *P* < 0.0001), while most dogs from which tropical lineage were collected (52/70; 74.3%) presented from June to November (*X*^*2*^ = 16.5, df = 1, *P* < 0.0001) (Fig. 2; Table 2). Adult *R. sanguineus* s.s. (temperate lineage) ticks were most commonly collected from dogs from March to August (1,453/1,578; 92.1%; 95% CI 90.6–93.3), while immature *R. sanguineus* s.s. (temperate lineage) ticks were almost all collected June–September (503/506; 99.4%; 95% CI 98.2–99.9) (Fig. 3a). Adult tropical lineage ticks were most commonly collected from dogs August through December (346/443; 78.1%; 95% CI 74.0–81.7), while immature tropical lineage ticks were almost all collected June–October (54/55; 98.2%; 95% CI 89.5–99.9) (Fig. 3b).

**Table 2.** Proportion of total *Rhipicephalus sanguineus* sensu stricto (temperate lineage) and tropical lineage of *R. sanguineus* sensu lato infestations identified on pets each month.

Month^*a*^	*R. sanguineus* s.s. (temperate lineage) only	*R. sanguineus* s.l. tropical lineage only	Mixed infestations
	*N* = 282	*N* = 70	*N* = 8
	*N* (%)	*N* (%)	*N* (%)
January	9 (3.2%)	1 (1.4%)	0
February	9 (3.2%)	5 (7.1%)	0
March	24 (8.5%)	3 (4.3%)	0
April	40 (14.2%)	5 (7.1%)	3 (37.5%)
May	30 (10.6%)	3 (4.3%)	0
June	41 (14.5%)	6 (8.6%)	2 (25.0%)
July	61 (21.6%)	4 (5.7%)	1 (12.5%)
August	43 (15.2%)	12 (17.1%)	0
September	16 (5.7%)	12 (17.1%)	1 (12.5%)
October	5 (1.8%)	11 (15.7%)	1 (12.5%)
November	2 (0.7%)	7 (10.0%)	0
December	2 (0.7%)	1 (1.4%)	0

^*a*^Month of collection was not reported for two pets infested with *R. sanguineus* s.s. (temperate lineage) and lineage was not identified for an additional 16 infested dogs.

## Discussion

The initial recognition that *R. sanguineus* s.l. comprises multiple tick species with similar morphology inspired subsequent efforts from researchers on several continents to understand the identity and diversity of lineages of *Rhipicephalus* spp. infesting dogs in a given region (Szabó et al. 2005, Burlini et al. 2010, Moraes-Filho et al. 2011, Nava et al. 2012, Dantas-Torres et al. 2013, Liu et al. 2013, Dantas-Torres et al. 2017, Hornok et al. 2017, Chitimia-Dobler et al. 2017, Chitimia-Dobler et al. 2019, Chandra et al. 2021, Páez-Triana et al. 2021, Mumcuoglu et al. 2022). In the United States, at least two lineages of *R. sanguineus* s.l. have been described: *R. sanguineus* s.s. (temperate lineage) in Arizona, California, Oklahoma, and Texas, and tropical lineage (referred to as *R. linnaei* by some authors) in Arizona, Florida, Hawaii, and southern Texas, with sympatric populations of the two lineages in some areas of Arizona and Texas (Eremeeva et al. 2011, Zemstova et al. 2016, Jones et al. 2017, Brophy et al. 2022). Results from the present study suggest that the *R. sanguineus* s.s. (temperate lineage) has a wider geographic distribution in the United States than previously recognized, with this tick identified on dogs or cats in 20 different states, although travel of pets may also lead to short-lived introduction of brown dog ticks to some premises. In addition, *R. sanguineus* s.s. (temperate lineage) ticks predominated among *R. sanguineus* s.l. infestations submitted by veterinarians, responsible for over three-quarters of the brown dog tick infestations identified from dogs and all the infestations found on cats in the present study. However, because ticks were collected passively and submissions were not balanced geographically, the apparent predominance of *R. sanguineus* s.s. (temperate lineage) in the present study may not accurately reflect the actual prevalence of each lineage throughout the United States.

Infestations with the tropical lineage of *R. sanguineus* s.l. were comparatively less common in the present study, and, when identified, largely restricted to the southernmost areas of the United States. Lineages of *R. sanguineus* s.l. appear to segregate according to elevation, latitude, and climatic factors (Zemtsova et al. 2016, Jones et al. 2017, Hornok et al. 2017, Sánchez-Montes et al. 2021, Brophy et al. 2022). For example, the tropical lineage has been found to persist primarily in areas with a mean average daily temperature >20°C, a limitation often attributed to the poor survivability of fed immature stages in harsher winter conditions (Zemtsova et al. 2016, Jones et al. 2017, Labruna et al. 2017, Saleh et al. 2021) and, in warmer countries, tropical may be the only lineage identified (Jones et al. 2017, Páez-Triana et al. 2021, Chandra et al. 2021). Accordingly, over 80% of the tropical lineage infestations identified in the present study were found on dogs in the warmest regions of the United States including southern California and southern Nevada, where this lineage has not been previously reported. However, we were surprised to also identify tropical lineage *R. sanguineus* s.l. from dogs from states with much cooler climates, including far northern states like Michigan, Minnesota, Washington (state), and Wisconsin, and confirmed that at least some of these likely resulted from recent travel of dogs with their owners. Recent travel history should always be a consideration when interpreting data on tick distribution based on ticks removed from companion animals or people, particularly when ticks are found in an unexpected region or at an unusual time of the year (Jones et al. 2017, Saleh et al. 2019, Xu et al. 2019, Eisen and Paddock, 2021; Brophy et al. 2022). The endophilic propensity of *R. sanguineus* s.l. may allow introduced populations to persist indoors for some period of time after introduction even if not ideally suited to a given climate (Dantas-Torres 2010).

Apparent differences in vector capacity between *R. sanguineus* s.s. (temperate lineage) and tropical lineages may be epidemiologically important and could influence where geographically infections with particular pathogens are more commonly diagnosed (Dantas-Torres and Otranto 2015). For example, experimental and epidemiologic data indicate that *E. canis*, causative agent of canine monocytic ehrlichiosis (CME), is primarily transmitted by tropical lineage *R. sanguineus* s.l. and thus CME cases are more commonly diagnosed in regions where the tropical lineage predominates (Beall et al. 2012, Moraes-Filho et al. 2015). Conversely, *Ehrlichia chaffeensis* and *Ehrlichia ewingii*, which share *Amblyomma americanum* as primary vector, more commonly infect dogs in states in the United States where *R. sanguineus* s.s. (temperate lineage) predominates and thus transmission of *E. canis* would be expected to be less common, although brown dog ticks (lineage not specified) have been implicated as a potential secondary vector of *E. chaffeensis* and *E. ewingii* in some regions (Ndip et al. 2007, Beall et al. 2012, Zemstova et al. 2016). Although more research is needed, some data suggest that other pathogens transmitted by *R. sanguineus* s.l., including *Rickettsia rickettsii*, *Anaplasma platys*, and *Hepatozoon canis*, may have similar affinity for transmission by only individual lineages within the *R. sanguineus* s.l. species complex (Sanogo et al. 2003, Inokuma et al. 2003, Eremeeva et al. 2011, Demoner 2013). The extent to which distinct lineages of *R. sanguineus* s.l. serve as important vectors for both canine and zoonotic disease agents warrants further investigation, particularly given the importance of brown dog tick transmitted *R. rickettsii* as a public health concern (Demma et al. 2005, Álvarez-Hernández et al. 2017).

The present paper also provides the first report in North America of 12S rRNA sequence consistent with an *R. sanguineus* s.l. lineage originally referred to as *Rhipicephalus* sp. I on three different dogs, including two from New Mexico and one from Idaho. This lineage (*Rhipicephalus* sp. I or ‘southeastern Europe lineage’) was originally identified in Italy and Greece and has since been described from eastern Europe and the Middle East (Dantas-Torres et al. 2013, Chitimia-Dobler et al. 2017, Chitimia-Dobler et al. 2019, Mumcuoglu et al. 2022). Although *Rhipicephalus* sp. I has been shown experimentally to successfully breed with *R. sanguineus* s.s. (temperate lineage, also *Rhipicephalus* sp. II), and limited sympatric populations have been described, the two temperate lineages (*R. sanguineus* s.s. and ‘southeastern Europe lineage’ *Rhipicephalus* sp. I) appear both genetically and geographically distinct (Chitimia-Dobler et al. 2017, Dantas-Torres et al. 2018, Chitimia-Dobler et al. 2019). Alternatively, our finding may be an artifact from detecting low frequency haplotypes in ticks with mitochondrial heteroplasmy (Dantas-Torres et al. 2018). Two of the three dogs with ‘southeastern Europe lineage’ *Rhipicephalus* sp. I temperate lineage ticks in the present study were also co-infested with a greater number of *R. sanguineus* s.s. (temperate lineage) ticks.

Regardless, the finding of a small number of *Rhipicephalus* sp. I or ‘southeastern Europe lineage’ ticks on dogs in the United States in the present study was unexpected and may represent introduction following travel much like our discovery of tropical lineage ticks on dogs in unexpected geographies (Fig. 1). Careful monitoring of tick populations is important to detect introductions when they occur, including ticks introduced from other continents (Beard et al. 2018). Tick populations in North America, as in other areas of the world, are continuing to expand, bolstered in part by warmer temperatures that support establishment once introductions occur (Minigan et al. 2018, Sonenshine 2018). Our findings support the interpretation that sympatric populations of *R. sanguineus* s.s. (temperate lineage) and tropical lineage are present in Arizona, southern Texas, and southern California, as has been suggested by others, although further research that includes environmental collections and full travel history on all dogs would be needed to confirm this assertion (Jones et al. 2017, Brophy et al. 2022). Indeed, four of the eight dogs found co-infested with both *R. sanguineus* s.s. (temperate lineage) and tropical lineage ticks in the present study were from the same household. Given the endophilic propensity of the *R. sanguineus* s.l. complex, multiple lineages can persist in the same region (Dantas-Torres et al. 2008). To limit further geographic spread, all dogs should be routinely treated with acaricides and especially when traveling between regions; this recommendation is particularly important when owners travel with pets to tropical climates during the cooler months when ticks may not be a major concern at home (Wright et al. 2020).

The seasonality of *R. sanguineus* s.l. in the United States is not well documented and largely based on historic work prior to recognition of distinct lineages (Koch 1982). Survivability likely depends on vulnerability of different stages to the elements in a given region although once indoor infestations of are established, *R. sanguineus* s.l. activity may be sustained year-round provided dogs are available as a food source (Dantas-Torres 2010). In warmer climates, *R. sanguineus* s.l. can also survive outdoors and are remarkably resistant to desiccation (Koch 1982, Dantas-Torres 2010). When outdoors, this species tends to seek refuge between rocks or in cracks and crevices in the ground and stony outcroppings; ovipositing females are found in these protective niches to ensure their safety and that of their progeny (Walker et al. 2000, Dantas-Torres 2010). Data from the present study suggests that, although both lineages were collected from pets throughout the year, *R. sanguineus* s.s. (temperate lineage) may be most active in spring and summer, when conditions in temperate areas are more hospitable, and that tropical lineage ticks are more likely to be found in late summer and fall. However, as has been noted in prior research, the passive sample collection strategy used limits drawing firm conclusions about seasonality (Brophy et al. 2022). If supported by subsequent, prospective studies of *R. sanguineus* s.l. activity, this difference may reflect the absence of a pronounced winter diapause in the tropical lineage and the presence of winter diapause in *R. sanguineus* s.s. (temperate lineage) (Labruna et al. 2017). Additional field work that carefully controls for recent introduction via travel from another region and consistent assessment of dogs throughout the year will be needed to confirm precise seasonal patterns. However, the present study indicates that both lineages are present on dogs year-round in the United States, supporting recommendations to maintain dogs on consistent tick control (Creevy et al. 2019).

One limitation of the present study was lack of complete sequence data for all ticks on heavily infested dogs. Previous research from our group did not reveal the presence of mixed *R. sanguineus* s.s. (temperate lineage) and tropical lineage infestations on dogs (Jones et al. 2017), but subsequent work by others in Arizona, USA, did occasionally detect *R. sanguineus* s.s. (temperate lineage) and tropical lineages from the same dog and premise (Brophy et al. 2022). Although most dogs in the present study were infested with only a single lineage of *R. sanguineus* s.l., sequencing multiple (*n* = up to 10) ticks from the same dog revealed occasional mixed infestations with both *R. sanguineus* s.s. (temperate lineage) and tropical lineage present, as well as diversity in 12S rRNA gene sequence of *R. sanguineus* s.s. (temperate lineage) as previously described (Jones et al. 2017, Brophy et al. 2022). Indeed, sequencing at least 5 ticks from each pet, when possible, revealed multiple 12S rRNA sequences of *R. sanguineus* s.s. (temperate lineage) in half of the infested pets in the present study (data not shown), supporting the interpretation that 12S rRNA sequence variation is common in this tick species. In addition, sequencing multiple ticks revealed *Rhipicephalus* sp. I, a southeastern Europe lineage, on two dogs that were also infested with *R. sanguineus* s.s. (temperate lineage) more commonly found in the United States (Dantas-Torres et al. 2013, Zemtsova et al. 2016, Jones et al. 2017, Brophy et al. 2022). Had we sequenced a larger number of ticks from these dogs, greater sequence diversity may have been identified.

Other limitations include the passive method of sample submission, an incomplete travel history for most dogs, and lack of information about acaricidal treatment of dogs. Passive or convenience sampling can result in under-recognition of infestations in general and may bias seasonality data if infestations are largely only identified during peak tick activity, thereby limiting documentation during other times of the year (Brophy et al. 2022). A complete recent travel history was not routinely collected and, when obtained, relied on owner recollection. We also did not have detailed records regarding contact with other dogs such as recent adoption from a shelter, dog day care attendance, or stay at a boarding kennel, factors which may increase both risk of infestation and propensity for mixed lineage infestations as *R. sanguineus* s.l. are acquired from environments with dogs (Dantas-Torres 2010). Treatment of dogs with acaricides, if any, was also not recorded and may have limited the number of *R. sanguineus* s.l. specimens available for sequencing from each infested pet.

Finally, because our goal was understanding biogeography and seasonality of the two main lineages rather than deep phylogenetic analysis, we chose to only sequence a 12S rRNA mitochondrial gene fragment in the present study. Research from other regions using ticks held in museum collections for over a century has shown that this marker (12S rRNA gene) is useful and stable over time and allows consistent identification of established lineages. Sequencing additional gene targets would not be expected to shift identification of tick lineage (i.e., *R. sanguineus* s.s. (temperate), tropical, or southeastern Europe *Rhipicephalus* sp. I), but such data would support more robust phylogenetic inquiry, particularly within lineages (Dantas-Torres 2010, Dantas-Torres et al. 2018, Chandra et al. 2021). In addition, sequence could not be successfully amplified from ticks from 16 dogs, likely due to nucleic acid degradation prior to submission; data from these infestations may have extended the known distribution and seasonal activity of the different lineages of *R. sanguineus* s.l. active in the United States. Nevertheless, the findings in the present study advance our knowledge of distribution of *R. sanguineus* s.s. (temperate lineage) and tropical lineage in the United States, suggest these two species may have different peak seasonal activity periods, and support the interpretation that, while uncommon, co-infestation with both lineages occasionally occurs, an important consideration for future research (Zemtsova et al. 2016, Jones et al. 2017, Brophy et al. 2022).
